# The role of public health services in implementing heat health action plans in Germany: Karin Geffert

**DOI:** 10.1093/eurpub/ckac130.070

**Published:** 2022-10-25

**Authors:** K Geffert, E Rehfuess, B Rechel

**Affiliations:** 1 Public Health and Health Services Research, Ludwig-Maximilians-Universität, Munich, Germany; 2 Pettenkofer School of Public Health, Ludwig-Maximilians-Universität, Munich, Germany; 3 European Observatory, LSHTM, London, UK

## Abstract

**Background:**

The rise in extreme heat periods is a major public health challenge of climate change and the World Health Organization therefore recommends the implementation of heat health action plans (HHAPs). In Germany, HHAPs are not implemented in a comprehensive manner nor nationwide. Public health authorities have been identified as key actors with regards to heat and health. This study aims at assessing the role of public health services in the implementation of HHAPs in Germany.

**Methods:**

First, a review of the scientific and grey literature on the role of public health services in heat adaptation in Europe was conducted. Second, a policy document analysis of the legislation of Germany's federal states for public health services and their potential role in the implementation of HHAPs was carried out. Finally, semi-structured interviews with selected experts from multiple sectors at the local, federal and national level on their perception of the role of public health services in the implementation of HHAPs in Germany were undertaken.

**Results:**

Preliminary findings show that the legal framework for public health services in the different federal states addresses environmental health and civil protection to varying extents, but that climate change-specific health risks are barely mentioned. The expert interviews revealed perceived barriers for the public health services to implement HHAPs, notably with regards to personnel (e.g. competencies, time), organizational structures (e.g. financial resources, administrative structures, legal mandates) and competing other tasks (e.g. COVID-19 response). Facilitators included motivated individuals, funding opportunities for cross-sectoral collaboration and political support.

**Conclusions:**

The role of public health services in HHAP implementation in Germany varies widely between the different geographic settings and is influenced by several factors at the individual, organizational and political level.

**Key messages:**

- Public health services can play an important role in heat adaptation, for example through coordinating the implementation of heat health action plans.
- Strong legal frameworks, sufficient organizational structures and well-trained personnel are important preconditions for an effective response by public health services to climate change challenges.

